# Comparative analyses of error handling strategies for next-generation sequencing in precision medicine

**DOI:** 10.1038/s41598-020-62675-8

**Published:** 2020-04-01

**Authors:** Hannah F. Löchel, Dominik Heider

**Affiliations:** Philipps-University of Marburg, Department of Mathematics and Computer Science, Marburg, 353032 Germany

**Keywords:** Data mining, Genetics

## Abstract

Next-generation sequencing (NGS) offers the opportunity to sequence millions and billions of DNA sequences in a short period, leading to novel applications in personalized medicine, such as cancer diagnostics or antiviral therapy. Nevertheless, sequencing technologies have different error rates, which occur during the sequencing process. If the NGS data is used for diagnostics, these sequences with errors are typically neglected or a worst-case scenario is assumed. In the current study, we focused on the impact of ambiguous bases on therapy recommendations for Human Immunodeficiency Virus 1 (HIV-1) patients. Concretely, we analyzed the treatment recommendation with entry blockers based on prediction models for co-receptor tropism. We compared three different error handling strategies that have been used in the literature, namely (i) neglection, (ii) worst-case assumption, and (iii) deconvolution with a majority vote. We could show that for two or more ambiguous positions per sequence a reliable prediction is generally no longer possible. Moreover, also the position of ambiguity plays a crucial role. Thus, we analyzed the error probability distributions of existing sequencing technologies, e.g., Illumina MiSeq or PacBio, with respect to the aforementioned error handling strategies and it turned out that neglection outperforms the other strategies in the case where no systematic errors are present. In other cases, the deconvolution strategy with the majority vote should be preferred.

## Introduction

Next-generation sequencing (NGS) offers the opportunity to sequence large amounts of DNA sequences in a short time with low costs^1,2^. Unfortunately, the different technologies exhibit different error rates between 0.01% and 15%^3^. While there are different error rates of the different sequencing technologies, the error rates are also affected by sequence composition, motifs, and other sequence-dependent effects, such as secondary structure formation^4,5^. Due to the reduced costs and huge amounts of sequence data that can be generated, NGS has entered clinical applications for personalized therapy. These applications include cancer diagnosis and therapy, e.g., there are several FDA-approved drugs for lung cancer, melanoma, leukemia, colon- and ovarian cancer, which are based on the results of NGS analyses^6^. While these approaches have entered clinical practice, there is however an important source of errors, including insertions, deletions, and substitutions within the sequencing process. Substitutions are the most important and most common error type, where one nucleotide is exchanged by another one or where the existing nucleotide could not be determined (i.e., ambiguity, typically shown as an N)^7^. These errors are of particular importance if the sequence is used, for instance, in diagnostics tests. There are several ways to handle these sequencing errors, however, neglection and worst-case scenario assumptions are the most frequently used ones. The worst-case scenario assumes that always the worst option, in terms of therapy, e.g., highly resistant mutation, is present. Neglection removes all those sequences in the NGS data which contain ambiguities, however, this could lead to a bias, if the error is not random but caused by the sequence itself, e.g., specific motifs. In contrast, the worst-case scenario assumption can lead to extremely conservative decisions, which could exclude patients from treatment who might benefit. Another error handling strategy is deconvolution with the majority vote, i.e., that the ambiguities are resolved, all combinations are predicted, and the majority prediction is considered. However, this strategy is computationally expensive in case of many ambiguous positions. For instance, one ambiguous position leads to four different sequences. However, sequences with $k$ ambiguous position lead to ${n}^{k}$ different sequences, with $n=4$, the four possible nucleotides A, C, G, and T. In the current study, we analyzed the effect of sequencing errors on diagnostic tests as well as the different error handling strategies. We focused on ambiguities since deletions also result in an ambiguous position in the DNA sequence and can, therefore, be handled like an ambiguity. Whereas the exact position of substitutions and inserts might not be easy to detect and have a high impact on protein-level. To this end, we investigated the impact of sequencing errors on the prediction of Human Immunodeficiency Virus 1 (HIV-1) tropism, which is important for HIV therapy with entry inhibitors, such as Maraviroc^8^. The entry of HIV-1 in the host cell is mediated by the binding of the gp120 surface protein, in particular, the V3-Loop, of HIV-1 to the CD4 receptor and a co-receptor (CCR5 or CXCR4)^9^. Several prediction tools are available, e.g., geno2pheno[coreceptor]^10^, T-CUP^11^, or PhenoSeq^12^. In order to avoid biases with respect to specific unknown sequences that have been used for training of existing tools, we used our tool T-CUP as baseline prediction, since it shows the best performance in comparison to other tools, and delivers a pseudo-probability^11^, where the training data is publicly available^13^ and which has been demonstrated to be reliable also for NGS data^14^. In contrast to existing tools such as geno2pheno[coreceptor]^10^ or PhenoSeq^12^, T-CUP offers high-throughput analyses in an automated manner, and is thus, also applicable to the huge amounts of sequences used in the current study.

## Results

**Figure 1:**
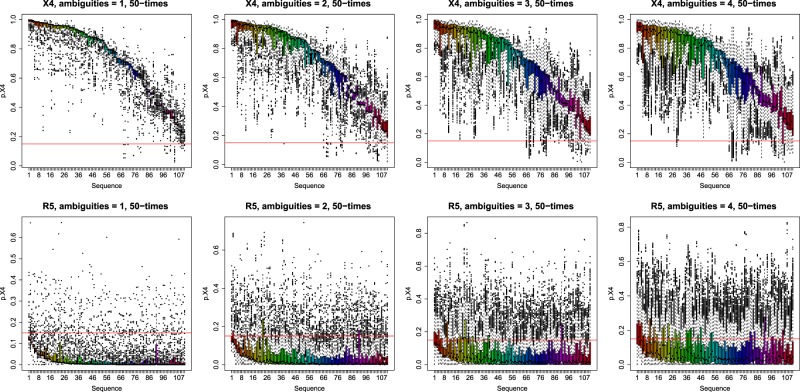
Boxplots for random ambiguities; sequences are ordered by decreasing original $p.X4$. The red line marks the cutoff for X4 prediction.

In Figure 1 the results for the randomly introduced ambiguities (one up to four) are shown for the X4 and R5, respectively. The sequences are ordered according to their pseudo-probability $p.X4$ for the original sequence, i.e., without ambiguities. The red line marks the cutoff for $p.X4$, i.e., those sequences that have a $p.X4\,\ge$ cutoff are considered as X4 and those with $p.X4\, <$ cutoff are considered as R5.

With increasing $k$, i.e., an increasing number of ambiguities per sequence, the variances in $p.X4$ increase significantly. However, those sequences that have a high $p.X4$, i.e., they have a high probability of being X4-tropic, are still predicted to be X4 even for $k > 1$.

**Figure 2:**
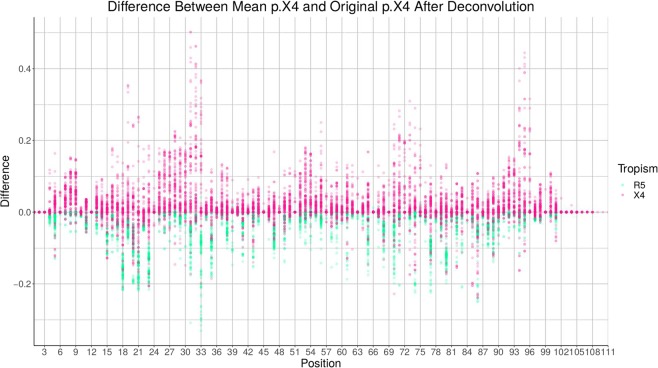
Difference for all positions. X4 and R5 are shown in pink and green, respectively.

**Figure 3:**
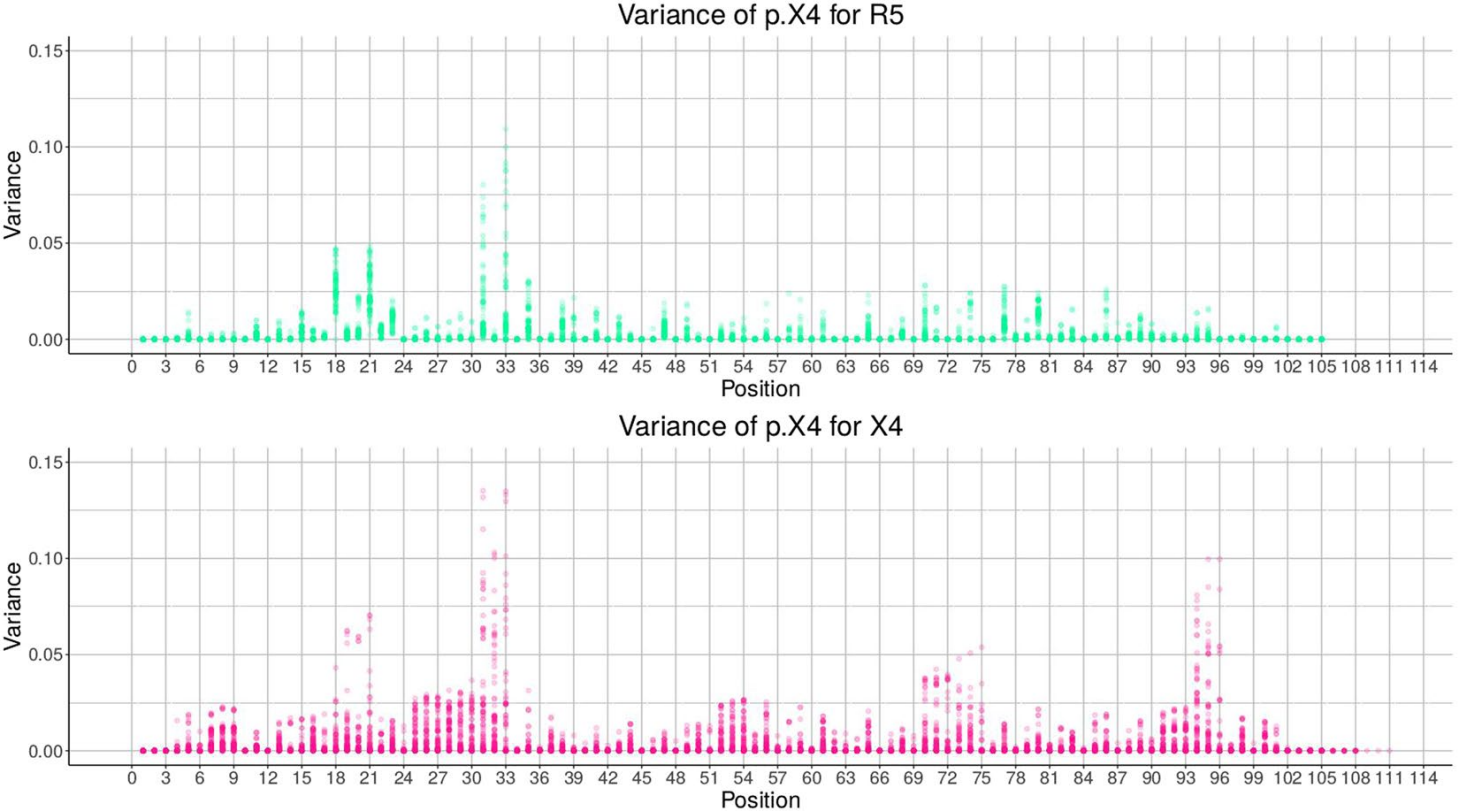
Variance for all positions. X4 and R5 are shown in pink and green, respectively.

In Figures 2 and 3 the results of the position-specific iterative replacements are shown (X4 pink, R5 green), i.e., the difference between mean and original $p.X4$ and the variance, respectively. Noteworthy, positions 33, 72, and 75 (i.e., positions 11, 24, and 25 in the V3 loop) stand out, which are known to be associated with tropism^15,16^.

**Figure 4:**
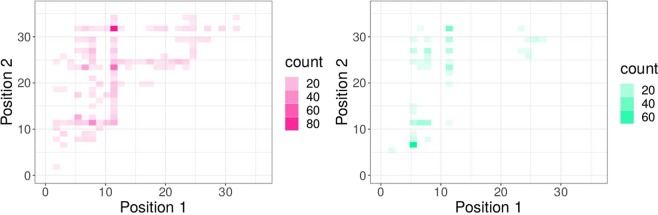
Pareto front for two iterative mutations. (**A**) X4; (**B**) R5.

**Figure 5:**
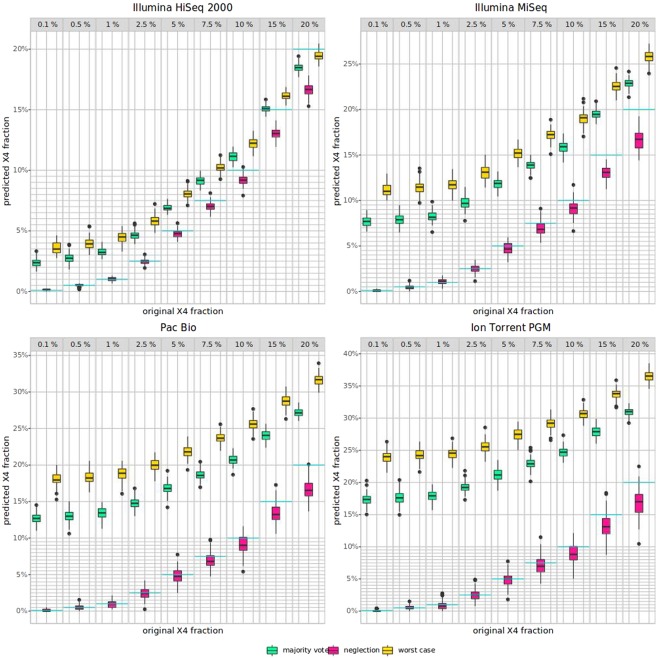
NGS-simulated data. Results for worst-case, deconvolution with majority vote, and neglection are shown in green, red, and yellow, respectively. The blue line marks the original X4 fraction.

**Figure 6:**
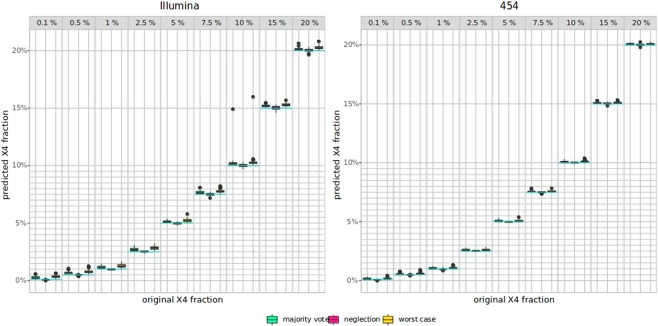
Position based NGS-simulated data. Results for worst-case, deconvolution with majority vote, and neglection are shown in yellow, green, and red, respectively. The blue line marks the original X4 fraction.

While Figures 2 and 3 show the impact of single mutations on $p.X4$, we also analyzed the co-occurrence of mutations by analysing the resulting Pareto front of two mutations (see Fig. 4). For the X4 dataset, position 11 in the V3 loop plays an important role for co-receptor tropism, more or less independently from the second mutation, however, in particular with position 24 or 31. For the R5 dataset, mutations in position 6 have a strong impact on $p.X4$, as well as mutations in combination with position 11, especially position 11 in combination with position 31. We also analyzed the error handling strategies (neglection, worst-case, and deconvolution) in a real-world setting, where we simulated next-generation sequencing data with error rates from different sequencing technologies. The results on the imbalanced data with different X4 proportions are shown in Fig. 5. It turned out that the neglection strategy works best in all analyzed technologies and X4 proportions, followed by deconvolution with the majority vote, both outperforming the worst-case scenario. Moreover, we analyzed position-specific error rates determined by * Raymond et al. (2017)*^17^ for Illumina MiSeq and 454 GS-Junior. In Fig. 6 the results for position-specific error rates are shown. The overall error rate is very low, however, the results are similar, but not as pronounced.

## Discussion

The occurrence of ambiguities can have a high impact on the results of prediction models and thus therapy recommendations. In our analyses, we used the prediction of HIV-1 tropism as an example for next-generation sequencing in precision medicine, where the tropism is determined based on V3 sequences. It turned out that ambiguities at some positions, especially positions 11, 24, and 25 (in accordance with the literature^18,19^) as single substitutions or in combination, can have a large effect on subsequent predictions. We compared the three different error handling strategies, namely neglection, worst-case, and deconvolution with the majority vote. It turned out that neglection outperformed the other approaches in our simulations based on random equally distributed errors in all sequencing technologies as well as in position-specific error distributions. Nevertheless, the effect was much smaller in the latter. Due to the fact that we had to limit our analyses to V3-sequences, we can only refer our results to those sequences. Further analyses have to be made to generalize our findings on error handling strategies to other targets, e.g., reverse transcriptase, protease, and integrase.

It is noteworthy that the neglection strategy is very restrictive and could lead to a loss of large amounts of data in case of systematic errors, e.g., sequence motifs or other sequence-dependent effects, such as secondary structure formation^4,5^, thus potentially introducing biases in the prediction and thus therapy recommendation. Therefore, it is reasonable to use the deconvolution strategy in cases where a huge fraction of reads in the data contains ambiguities. The worst-case strategy, however, performs worse compared to both other strategies and there is no scenario where this strategy seems to be reasonable. A recent study suggests that lowering the threshold of resistance testing might come with a reduction of specificity in HIV-1 resistance testing, but increases the identification of people at risk of virological failure, i.e., sensitivity^20^. So far there are cases where patients experienced therapy failure during a co-receptor antagonist, while the X4 ratio was beneath the threshold of phenotypic testing^11^. Depending on the applied error-strategy the threshold for a decision might be exceeded, e.g., by using the worst-case-scenario for low X4-ratio, which could then, in turn, lead to the wrong treatment decision.

## Methods

### Dataset

**Figure 7:**
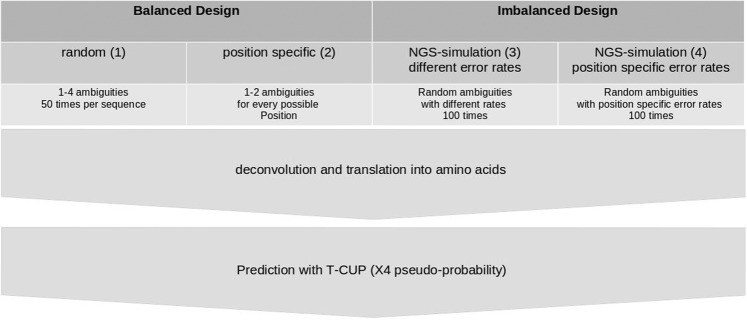
Workflow. We generated ambiguous DNA sequences in four different ways, created all possible sequences, and translated them into protein sequences. The protein sequences were used to predict the X4 pseudo-probability with the R package T-CUP. In the balanced design (1) with one up to four random ambiguities and (2) position-specific in an iterative manner with one up to two ambiguities, and in an imbalanced design with different X4 fractions with different error rates of the NGS-sequencers (3) and (4) with position-specific error rates for V3 sequences for different NGS-sequencers.

We collected sequence data from the genomic region of the V3 of subtype B HIV-1 viruses from the Los Alamos HIV Database (http://hiv-web.lanl.gov) in March 2017. We used the R package seqinr^21^ to clean the raw data concerning ambiguities, leading to a dataset of 3,580 R5- and 214 X4-strains (see Table 1). The sequences were translated into protein sequences using seqinr and subsequently predicted with T-CUP^11^. Only those sequences that were correctly predicted (R5: $3,395$, i.e., specificity $=\,94.83 \%$ and X4: 207, i.e., sensitivity $=\,96.73 \%$) by T-CUP were used in further analyses (see Fig. 7). Moreover, unique sequences were kept, leading to a final dataset of 1,597 R- and 111 X4-viruses.

**Table 1:** Number of sequences after different preprocessing steps.

Dataset	Los Alamos HIV Database	after cleaning	correctly predicted	unique
R5	3585	3580	3395	1597
X4	214	214	207	111

For our evaluations, we simulated a balanced design with an equal number of R5 and X4 to avoid a class bias. To this end, we randomly selected 111 sequences from the R5 dataset. Table 2 shows the number of analyzed sequences for the balanced designed datasets. Depending on the number of substitutions, up to more than 3 million sequences are generated for further analyses. After translation into protein sequences, this corresponds to more than 2.5 million different protein sequences in total that have been predicted with T-CUP only for the balanced design. Nevertheless, we also created datasets with different X4 proportions (see Table 3). We used the whole amount of unique R5-sequences and randomly selected X4-sequences with replacement to reach the given percentage.

**Table 2:** Number of sequences after deconvolution with varying number of ambiguities with random (1–4) or position-specific substitutions (1–2).

Method	On DNA level	X4 sequences	R5 sequences
1 random substitution	44,400	21,442	21,482
2 random substitutions	177,600	48,138	49,810
3 random substitutions	710,400	146,383	149,287
4 random substitutions	2,841,600	419,693	442,608
1 position specific substitution	37,864	11,598	11,616
2 position specific substitutions	3,180,789	600,306	602,043

**Table 3:** X4 portions for simulated data.

	0.1 %	0.5 %	1 %	2.5 %	5 %	7.5 %	10 %	15 %	20 %
R5	1,597	1,597	1,597	1,597	1,597	1,597	1,597	1,597	1,597
X4	2	8	16	40	80	120	160	240	319

### Analyses of error handling strategies

To analyze the behavior of the three error handling strategies, namely (i) neglection, (ii) worst-case, and (iii) deconvolution with the majority vote, we used two different approaches for introducing ambiguity in the balanced design. First, we used a general statistical approach based on the whole sequence, and second, we used a position-specific approach (see Fig. 7). In the statistical approach, we randomly introduced (50 repetitions) one up to four ambiguities per sequence. For the position-based approach, we introduced ambiguities for every single position iteratively to analyze position-specific effects. For both approaches, we analyzed the impact of these ambiguities on tropism prediction, e.g., by estimating the variance (var) for $p.X4$ for each position and the difference (diff) of the mean and original prediction (i.e., without ambiguities).

$${\rm{var}}=\frac{1}{n}\mathop{\sum }\limits_{i=1}^{n}{x}_{i}-\bar{x}$$

$${\rm{diff}}=p.X{4}_{original}-p.X{4}_{mean}$$

Furthermore, we calculated the Pareto maxima of variance and difference for each position with the R package rPref^22^. For the imbalanced data, i.e., the datasets with different X4-proportions (ranging from $0.1 \%$ to $20 \%$), we analyzed different rates of substitutions.

To analyze the error handling strategies in a real-world setting, we simulated next-generation sequencing data with real error rates from different technologies, including Illumina, PacBio, and Ion Torrent (see Table 4) using selection and replacement from our dataset. Moreover, we analyzed position-specific error rates determined by Raymond *et al*.^17^ for V3 sequences for Illumina MiSeq and 454 GS-Junior. In total, we generated and analyzed over 80 million protein sequences in our analyses.

**Table 4:** Error rates.

Method	Error type	Error rate
Illumina HiSeq 2000	of all types	0.26%^1^
Illumina MiSeq	of all types	0.8%^1^
PacBio CCS	Missmatch	1.30%^23^
Ion Torrent PGM	of all types	1.71%^1^
